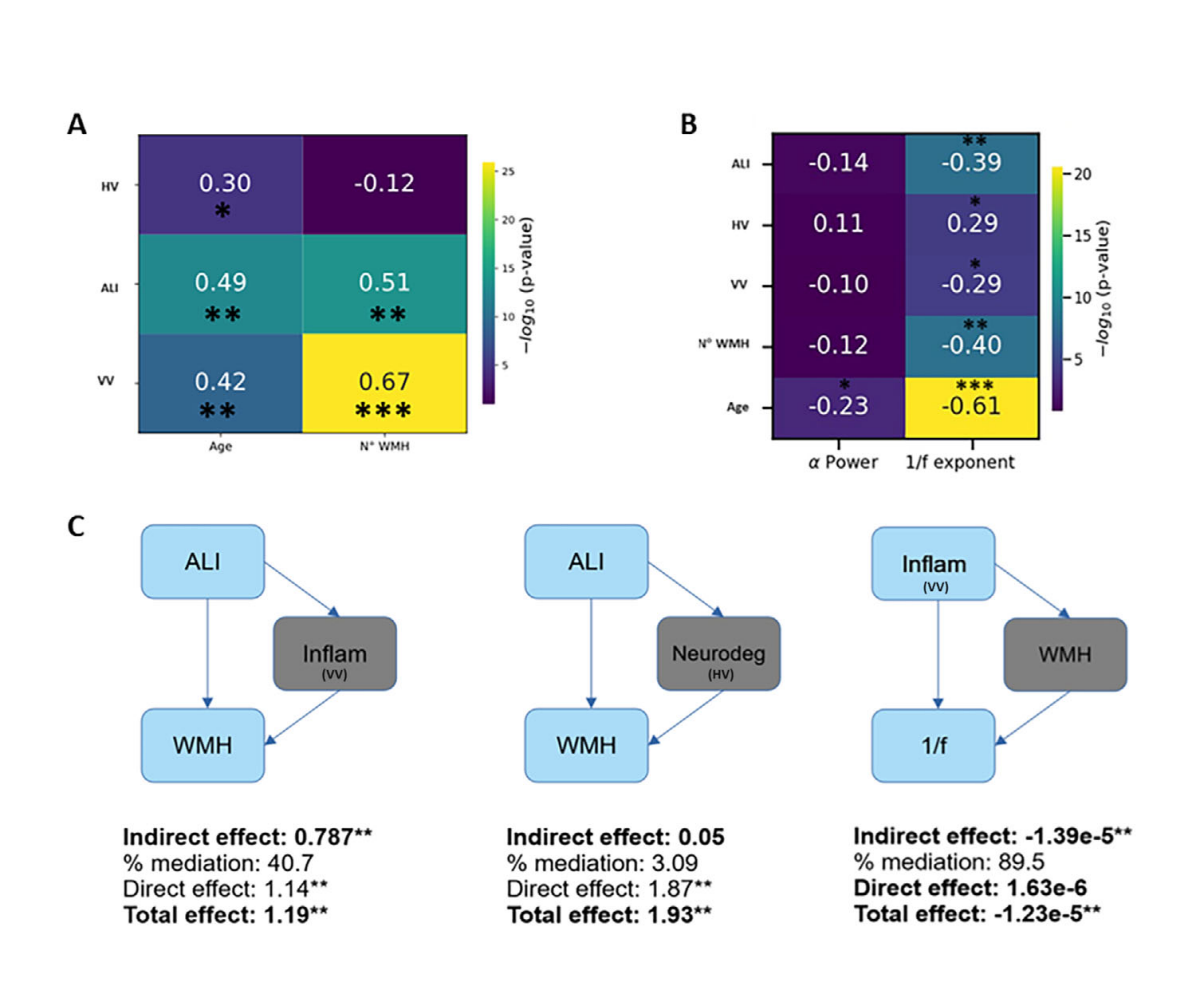# Allostasis links white matter hyperintensities with age‐related human brain structure and function: evidence from EEG and structural MRI

**DOI:** 10.1002/alz.093764

**Published:** 2025-01-09

**Authors:** Daniel Franco O'Byrne, Ana Maria Castro Laguardia, Carolina Delgado, Cecilia Gonzalez Campo, Agustín Ibáñez, Vicente Medel

**Affiliations:** ^1^ Latin American Brain Health Institute (BrainLat), Universidad Adolfo Ibáñez, Santiago, Región Metropolitana Chile; ^2^ Center for Social and Cognitive Neuroscience, Adolfo Ibañez University, Santiago, Región Metropolitana Chile; ^3^ Universidad de Chile, Santiago, Region Metropolitana Chile; ^4^ CONICET, Buenos Aires Argentina

## Abstract

**Background:**

The human brain integrity relies on the synergistic interplay between neural activity and supporting vascular and metabolic processes throughout life. This relationship, ruled by allostatic mechanisms, regulates brain architecture and activity. White matter hyperintensities (WMH) serve as indicators of the vascular impact on brain structure. While the strong association between vascular/metabolic systems and brain structure/function is established, the precise mechanisms linking allostasis to WMH remain elusive. We hypothesized that allostasis influences white matter through neuroinflammatory mechanisms, and WMH mediate neuroinflammatory effects on EEG measures.

**Method:**

To test this hypothesis we conducted a comprehensive analysis on MRI, EEG, and multisystemic data from 196 subjects (20 to 75 years, 69 females) sourced from the LEMON database in Leipzig, Germany. Using the lesion segmentation tool (LST) on SPM12, we automatically identified WMH in fluid‐attenuated inversion recovery (FLAIR) images. FastSurfer was employed to determine the volumes of the bilateral hippocampus (HV) and lateral ventricles (VV), representing proxies for neurodegenerative and neuroinflammatory processes, respectively. Volumetric corrections were applied using total intracranial volume. Furthermore, a regression analysis was conducted to discern and subtract the shared contributions of VH and VV on each other. EEG data underwent spectral analysis for oscillatory (alpha waves) and aperiodic (1/f) components using NeuroDSP and FOOOF, respectively. An allostatic load index (ALI), incorporating lipid profile, inflammation, metabolic factors, and blood pressure, was computed. All measures were age, sex, and education level‐corrected.

**Result:**

We found age differences in every metric of interest, but importantly just ALI, VV and 1/f correlated with WMH number and those effects survived age correction (Figure 1 A and B). VV, but not HV mediates the relationship between ALI and WMH. Also, WMH completely mediate the effects of neuroinflammation over the 1/f (Figure 1 C).

**Conclusion:**

Our findings reveal that allostasis affects white matter through neuroinflammatory pathways, with WMH serving as crucial mediators of neuroinflammatory impact on EEG measures. This research provides key insights into the intricate interplay of vascular and metabolic factors with brain structure. Identified relationships and mediators open avenues for targeted interventions and personalized treatments in neurodegenerative and neuroinflammatory conditions.